# Post-Exercise Nasal Patency, Spirometry, and CBCT-Derived Lund–Mackay Scores in Competitive Swimmers: A Multivariate Analysis

**DOI:** 10.3390/jcm15155868

**Published:** 2026-07-27

**Authors:** Małgorzata Stachowicz, Mateusz Żbikowski, Aleksandra Stachowicz, Anna Kęska, Michał Stachowicz, Grzegorz Wójcik

**Affiliations:** 1 Department of Water and Winter Sports, Józef Piłsudski University of Physical Education in Warsaw, ul. Marymoncka 34, 00-968 Warsaw, Poland; 2 Department of Intelligent Systems and Data Science, Polish–Japanese Academy of Information Technology, ul. Koszykowa 86, 02-008 Warsaw, Poland; 3 Doctoral School, Józef Piłsudski University of Physical Education in Warsaw, ul. Marymoncka 34, 00-968 Warsaw, Poland; 4 Collegium Medicum, Nicolaus Copernicus University in Toruń, ul. Jagiełłońska 13–15, 85-067 Bydgoszcz, Poland; michal.stachowicz23@gmail.com; 5 Department of Neuroinformatics and Biomedical Engineering, Institute of Computer Science, Maria Curie-Sklodowska University in Lublin, ul. Akademicka 9/509, 20-033 Lublin, Poland

**Keywords:** exercise physiology, nasal patency, cone-beam CT, swimming, peak nasal inspiratory flow, sinonasal disease, tethered force, athletes

## Abstract

**Background/Objectives:** Competitive swimmers present simultaneously with supranormal lung function and a high prevalence of upper-airway structural pathology. This study examined whether CBCT-quantified sinus mucosal burden and post-exercise peak nasal inspiratory flow (PNIF) are related, and whether their combination with spirometry identifies distinct ventilatory subgroups, in competitive swimmers. **Methods:** Twenty-nine competitive swimmers (18 male, 11 female; age 20.7 ± 2.8 yr) underwent nasal endoscopy (*n* = 29), CBCT with Lund–Mackay (LM) scoring (*n* = 20), bilateral PNIF at rest (T3) and ≤60 s post-sprint (T4), spirometry (FVC, FEV_1_, PEF, FEF_25–75_; GLI-2012 norms), and a 30 s maximal tethered sprint (Fmax, N). Principal component analysis (PCA) and hierarchical cluster analysis were applied to ΔPNIF, LM score, resting spirometry, and Fmax. **Results:** LM score and ΔPNIF were not significantly correlated (Spearman ρ = 0.32; *p* = 0.17). PCA yielded two components (63.1% of variance) that did not cleanly separate a mucosal-reactivity dimension from ventilatory reserve: LM score loaded together with Fmax on the second component, while ΔPNIF loaded moderately on both components. Hierarchical clustering identified three subgroups (silhouette coefficient 0.38), but none combined reduced ΔPNIF with elevated LM score; the subgroup with the highest mean LM score (*n* = 7) showed the highest mean ΔPNIF (+29.3 L/min) of the three. Resting spirometry was supranormal throughout (FEV_1_% 115.3 ± 14.2%) and did not differ between subgroups. Exercise-induced bronchodilation was pronounced across the cohort (ΔFEV_1_ +9.6 pp; d = 0.84), and tethered force (Fmax) was the sole correlate of World Aquatics performance (r = 0.560; *p* = 0.002). **Conclusions:** In this cohort, CBCT-derived sinus mucosal burden and post-exercise nasal decongestion were not significantly associated, and clustering did not identify a subgroup analogous to a “dysfunctional” ventilatory–sinonasal phenotype. Post-exercise PNIF and resting spirometry appear to capture largely independent aspects of exercise physiology in competitive swimmers, but neither, alone or combined with LM score, discriminated a distinct clinical subgroup in this sample. Tethered force remains the strongest correlate of competitive performance identified.

## 1. Introduction

Competitive swimmers develop supranormal pulmonary function, with forced vital capacity (FVC) and forced expiratory volume in one second (FEV_1_) typically reaching 110 to 125 percent of GLI-2012 reference values—a well-established physiological adaptation resulting from aquatic training [1,2]. At the same time, the chlorinated pool environment exposes the nasal and paranasal sinus mucosa to chronic inflammatory stimuli. High prevalences of nasal septum deviation (DNS), inferior turbinate hypertrophy (ITH), allergic rhinitis (AR) and mucosal changes within the paranasal sinuses have been reported in competitive swimmers [3,4]. Several mechanisms plausibly link the sport itself to this burden: repetitive flip-turn impacts and turbulent water entry during starts and turns expose the nasal septum and turbinates to repeated mechanical microtrauma, while chronic mucosal exposure to chlorine and its by-products (chloramines) is a recognised irritant and sensitising stimulus that can promote both non-allergic and allergic mucosal inflammation [4]. This creates an apparent paradox in which excellent ventilatory capacity coexists with widespread pathology of the upper airways. Clarifying whether this upper-airway burden translates into a functionally relevant, exercise-induced deficit—rather than remaining a purely structural finding of uncertain consequence—is clinically relevant for sports-medicine practice: if a subset of athletes could be shown to combine radiological sinus disease with impaired post-exercise nasal decongestion, this would identify a group who might benefit from targeted otolaryngological review that resting spirometry alone would not flag.

Beyond this general paradox, sinonasal complaints represent one of the most prevalent yet under-recognised health burdens in competitive athletes, and aquatic-sport participants in particular. Systematic reviews report rhinitis prevalence rates that substantially exceed those of the general population in elite athletes, with swimmers accumulating uniquely high chlorine and cold, dry air mucosal exposure during training [5]. Nasal obstruction in this population is frequently multifactorial, encompassing structural abnormalities, allergic and non-allergic rhinitis, and training-induced mucosal remodelling, and it meaningfully impairs quality of life and perceived respiratory comfort during competition [6,7]. Despite this burden, nasal symptoms and mild nasal trauma are often under-reported by athletes and under-recognised by sports-medicine staff, in part because they rarely produce measurable resting spirometric abnormality [8,9]. Sinonasal disease has also been mechanistically linked to exercise itself, with training-related mucosal inflammation acting as both a plausible cause and a consequence of impaired nasal function during exertion [10].

The clinical and physiological consequences of this paradox remain poorly understood. Most studies have characterised either lung function [1,2] or upper-airway pathology [3,4] in isolation, rarely linking them through an objective measure of airway function under exercise conditions. Peak nasal inspiratory flow (PNIF) measured after high-intensity exercise provides a functional index of sympathetically mediated nasal mucosal vasoreactivity, offering information about upper-airway function that complements, rather than duplicates, spirometric assessment of the lower airways [11,12]. PNIF is inexpensive and portable, and has been validated against acoustic rhinometry and rhinomanometry as a reliable screening tool for nasal obstruction across normal and clinical populations [13,14,15,16], making it particularly attractive for field-based assessment in athletic settings where laboratory-grade instrumentation is impractical.

Similarly, the Lund–Mackay (LM) CT score provides an objective, observer-independent measure of sinus mucosal burden [17] that predicts symptom severity and treatment response in chronic rhinosinusitis populations [18], yet is essentially absent from the sports physiology literature. Cone-beam CT (CBCT) offers diagnostic resolution comparable to conventional CT for paranasal sinus imaging at substantially lower radiation exposure [19], a practical advantage for athlete populations in whom repeated imaging may be considered. The nasal mucosa itself is richly vascularised and under strong autonomic control [20], providing the physiological basis for the rapid, exercise-induced vasomotor changes that PNIF is designed to capture.

Whether integrating CBCT-derived LM scores, post-exercise ΔPNIF, and spirometric data reveals phenotypically distinct respiratory profiles in competitive swimmers has not been studied. The concept of respiratory phenotyping has been productively applied in asthma and COPD research [21,22] but never in elite athletic populations. We tested three pre-specified hypotheses: (i) that ΔPNIF and LM score load on a single mucosal-reactivity component separable from ventilatory reserve; (ii) that data-driven clustering identifies phenotypically distinct groups invisible to resting spirometry; and (iii) that a multivariate model combining ΔPNIF and LM score could discriminate a subgroup combining elevated sinus mucosal burden with reduced post-exercise nasal decongestion (target discriminative performance: AUC ≥ 0.80).

## 2. Materials and Methods

### 2.1. Participants

Twenty-nine competitive swimmers (18 male, 11 female; age 20.7 ± 2.8 yr; range 18–34 yr) training at the intercollegiate competitive level (≥10 sessions/week) were recruited from AZS AWF Warsaw, the university sports club of the Józef Piłsudski University of Physical Education in Warsaw (Poland), whose swimming section competes in national intercollegiate leagues. Stroke specialisations: freestyle (*n* = 18), breaststroke (*n* = 6), butterfly (*n* = 4), backstroke (*n* = 1); given these small subgroup sizes, stroke-specific comparisons were not performed and are not reported. Active competitive status was defined as current registration with the Polish Swimming Federation (Polski Związek Pływacki, PZP), membership in a competitive swimming section, and active participation in competitions. Inclusion criteria: active competitive status as defined above, age ≥ 18 yr, no acute upper respiratory infection within 4 weeks. Exclusion criteria: current smoking or history of smoking, inhaled corticosteroids or systemic vasoconstrictors within 4 weeks, and any diagnosed cardiopulmonary or otolaryngological comorbidity other than the nasal/sinus conditions assessed as study outcomes.

### 2.2. Experimental Design

All measurements were performed in a single session in fixed order: (1) SNOT-22, NOSE-POL, and VAS questionnaires; (2) rigid nasal endoscopy ± CBCT; (3) 15 min seated rest; (4) resting PNIF (T3) and spirometry; (5) standardised aquatic warm-up; (6) 30 s tethered sprint with Fmax recording; (7) post-sprint PNIF (T4, ≤60 s) and spirometry (≤3 min). The SNOT-22, NOSE-POL, and VAS questionnaires served solely as clinical screening instruments to help identify participants warranting CBCT referral (together with endoscopic findings); they were not used as quantitative variables in the multivariate analyses reported below. CBCT was performed in 20 participants with clinical indications established during endoscopy or by SNOT-22 score. The threshold for CBCT referral was established a priori in agreement with the collaborating otolaryngologists: only participants judged by the examining physician, on the basis of the SNOT-22/NOSE-POL/VAS questionnaires together with the endoscopic examination, to show evidence of impaired nasal patency or another sinonasal abnormality at this initial assessment stage were referred for CBCT; the decision that CBCT was clinically warranted rested with the examining physician, informed by these questionnaire and endoscopic findings, rather than on a fixed numerical cut-off applied uniformly to all participants. Because CBCT was offered only to participants with a clinical indication rather than to the whole cohort, the 20 participants who underwent CBCT do not constitute a random sample of the 29 swimmers; this non-random ascertainment is expected to enrich the CBCT subgroup for sinonasal pathology relative to the full cohort and is addressed further in Section 2.8 and in the Section 4.

### 2.3. PNIF Measurement

PNIF was used as a functional surrogate of nasal airflow rather than a direct measure of mucosal congestion; it reflects the net effect of nasal patency, including mucosal vascular tone, on peak inspiratory flow. Bilateral PNIF was measured using an In-Check DIAL device (Clement Clarke International, Harlow, UK). Participants stood upright, exhaled fully, then performed a single maximal nasal inspiration; the best of two efforts per time point was recorded. ΔPNIF = T4 − T3 (L/min); values > 0 indicate exercise-induced nasal decongestion; values ≤ 0 indicate paradoxical congestion or absent decongestion [12].

### 2.4. Spirometry

Spirometry was performed using a Spirolab III spirometer (MIR, Medical International Research, Rome, Italy), according to ATS/ERS 2019 standards at rest and ≤3 min post-sprint. Parameters: FVC, FEV_1_, FEV_1_/FVC, PEF, FEF_25–75_, all expressed as percentage (%) of GLI-2012 predicted values [23,24]. Post-sprint increments (Δ, percentage points [pp]) were computed as post-sprint minus resting values.

### 2.5. CBCT and Lund–Mackay Scoring

Cone-beam CT (CBCT; Kodak 9600, Carestream Health, Rochester, NY, USA; 120 kV, 5 mA, 24 s exposure, dose index 1592 mGy·cm) was evaluated using the Lund–Mackay (LM) scoring system (range 0–24) by two independent, blinded observers [17]. An LM score of zero indicates absence of mucosal disease, values from 1 to 4 indicate mild disease, 5 to 9 moderate disease, and 10 or higher advanced disease, consistent with the staging conventions of the European Position Paper on Rhinosinusitis and Nasal Polyps [25].

### 2.6. Tethered Sprint and Force Measurement

A 30 s all-out tethered sprint was performed in a 25 m chlorinated indoor pool (water temperature 27–28 °C) using the swimmer’s primary stroke. Peak tethered force (Fmax, N) was recorded continuously via a MAX-6 dynamometric system (Staniak, Warsaw, Poland) with WTP-003 amplifier [26].

### 2.7. Laryngological Assessment

Rigid nasal endoscopy (0°, 4 mm; Karl Storz, Tuttlingen, Germany) classified DNS by the Mladina system (Types I–V) [27] and identified ITH (unilateral/bilateral), concha bullosa, and septum perforation. Confirmed AR required compatible clinical history and positive allergy testing.

### 2.8. Statistical Analysis

All analyses were performed in Python 3.12 (scikit-learn 1.4, SciPy 1.11, statsmodels 0.14). To test the hypotheses outlined in the Introduction, we applied principal component analysis (PCA) and hierarchical cluster analysis; these hypotheses were formulated prior to data collection but were evaluated in a single, moderately sized cohort without an a priori power calculation, so the analysis is best characterised as hypothesis-generating and exploratory rather than confirmatory. No a priori sample size or power calculation was performed; the cohort size was determined by the availability of competitive swimmers meeting inclusion criteria. Statistical significance was set at α = 0.05 throughout.

CBCT/Lund–Mackay (LM) data were verified at the case level against original radiology source records prior to analysis to ensure accurate participant-level linkage. Inter-observer reliability between the two independent LM readers, expressed as the intraclass correlation coefficient (ICC), was 0.94 (95% CI: 0.86–0.98), indicating excellent agreement. Median imputation of the LM score was chosen as a conservative, non-informative substitute for the 9 non-CBCT participants, who had unremarkable endoscopic and SNOT-22 findings and were for that reason not referred for CBCT; because CBCT ascertainment was non-random (Section 2.2), the measured LM scores likely over-represent sinonasal pathology relative to the full cohort.

PCA was applied to a standardised (z-score) matrix of six variables: ΔPNIF, LM score (imputed as described above), FEV_1_% rest, FVC% rest, PEF% rest, and Fmax. Allergic rhinitis status could not be reconstructed as a reliable, structured per-participant binary variable from the available source records and was therefore not included as a PCA input in this analysis; this six-variable-to-29-participant ratio (≈1:5) remains below conventional recommendations for stable PCA and cluster solutions, and the resulting components and clusters should be interpreted with this constraint in mind. Components were retained by the Kaiser criterion (eigenvalue > 1) and scree plot.

Hierarchical clustering used Ward’s minimum-variance linkage on PC1 and PC2 scores; optimal cluster number was determined by the dendrogram and silhouette analysis. The stability of this cluster solution to resampling was not formally tested.

Between-group comparisons of continuous variables were assessed using one-way ANOVA with Tukey’s post hoc test, or the Kruskal–Wallis test when normality (Shapiro–Wilk) was not met. Associations between continuous variables were assessed using Pearson and/or Spearman correlation coefficients, as appropriate. Paired *t*-tests compared rest vs. post-sprint values with Cohen’s d as effect size.

## 3. Results

### 3.1. Participant Characteristics and ENT Burden

Demographic and baseline physiological data are presented in Table 1. Males were taller (185 ± 5 vs. 174 ± 4 cm; *p* < 0.001) and generated significantly higher Fmax (236.9 ± 37.9 vs. 159.5 ± 18.2 N; *p* < 0.001). World Aquatics scores did not differ by sex (*p* = 0.45). DNS was present in 25/29 swimmers (86%; Mladina type II predominated, *n* = 12, 41%); ITH in 8/29 (27%); concha bullosa in 5/29 (17%); confirmed AR in 5/29 (17%). CBCT showed LM ≥ 1 in 9/20 (45%) and LM ≥ 10 in 3/20 (15%). Only 3/29 (10%) had no otolaryngological pathology. Resting spirometry was supranormal: FVC% = 118.1 ± 15.8%, FEV_1_% = 115.3 ± 14.2%, PEF% = 103.4 ± 26.6%, FEF_25–75_% = 123.0 ± 16.2%.

### 3.2. Whole-Group Ventilatory Response to 30 s Tethered Sprint

Sprint-induced ventilatory changes are presented in Table 2 (see also Figure S1). PNIF rose significantly (ΔPNIF = +24.5 L/min; 95% CI: 17.6–31.3; d = 1.36; *p* < 0.0001). FEV_1_% increased by +9.6 pp (d = 0.84; *p* < 0.0001), PEF% by +15.4 pp (d = 0.59; *p* = 0.004), and FEV_1_/FVC% by +5.4 pp (*p* = 0.014). The disproportionate rise in FEV_1_ relative to FVC is consistent with exercise-induced bronchodilation (EIBd) [28] mediated by β_2_-adrenergic activation of large-airway smooth muscle. FEF_25–75_ did not change significantly (*p* = 0.130). EIBd (FEV_1_ increase ≥5% predicted) was observed in 24/29 swimmers (83%).

Four swimmers (13.8%) showed paradoxical or absent post-exercise nasal decongestion (ΔPNIF ≤ 0): one with confirmed perennial AR (ΔPNIF = −20 L/min) and three with ΔPNIF = 0. Of the three with a directly measured LM score, all had low values (0–2); the fourth did not undergo CBCT. None met the LM ≥ 10 threshold used elsewhere in this manuscript to denote advanced sinus disease. Individual swimmer trajectories for all measures are shown in Figure S3.

### 3.3. Principal Component Analysis

**Figure 1:**
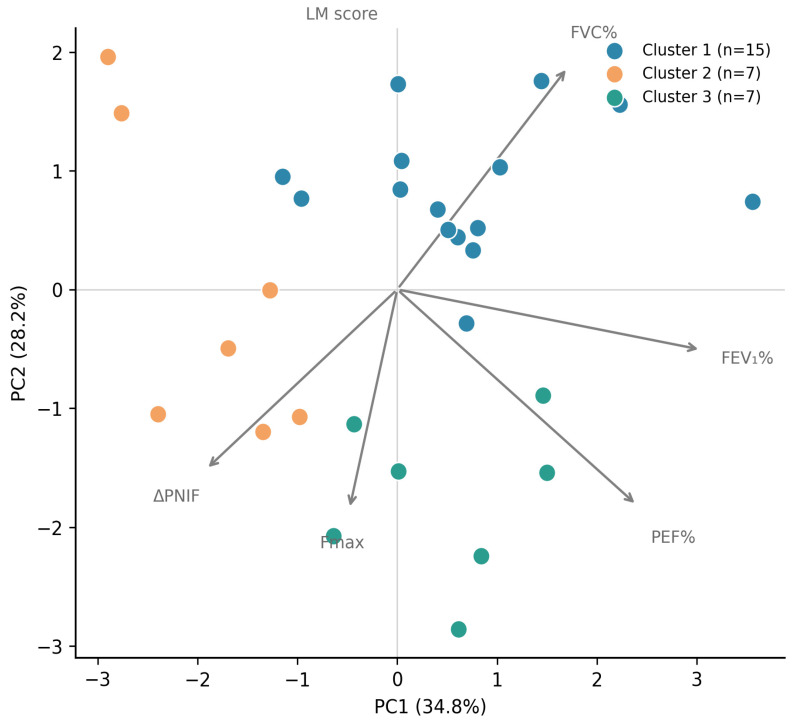
PCA biplot (*n* = 29). Points represent individual swimmers projected onto PC1 (34.8%) and PC2 (28.2%). Grey arrows: loading vectors. Colour coding: Cluster 1 (blue, *n* = 15), Cluster 2 (orange, *n* = 7), Cluster 3 (green, *n* = 7). ΔPNIF, change in peak nasal inspiratory flow (L/min); LM, Lund–Mackay score; Fmax, peak tethered force (N).

PCA of six standardised variables (ΔPNIF, LM score, resting FEV_1_%, FVC%, PEF%, and Fmax; allergic rhinitis status could not be reliably reconstructed as a structured per-participant variable and was not included, see Section 2.8) yielded two components with eigenvalues > 1, jointly explaining 63.1% of total variance (Table 3; Figure 1). PC1 (34.8% of variance) loaded most strongly on resting spirometric parameters (FEV_1_% +0.65, PEF% +0.51, FVC% +0.36) and moderately, negatively, on ΔPNIF (−0.41); LM score and Fmax loaded weakly on PC1 (|loading| ≤ 0.10). PC2 (28.2%) loaded on LM score (+0.53), Fmax (−0.44), FVC% (+0.44), PEF% (−0.43), and ΔPNIF (−0.36). Contrary to our a priori hypothesis, ΔPNIF and LM score did not load together on a single, cleanly separable mucosal-reactivity component: LM score’s strongest loading was on PC2, alongside Fmax, rather than with ΔPNIF.

### 3.4. Hierarchical Cluster Analysis

**Figure 2:**
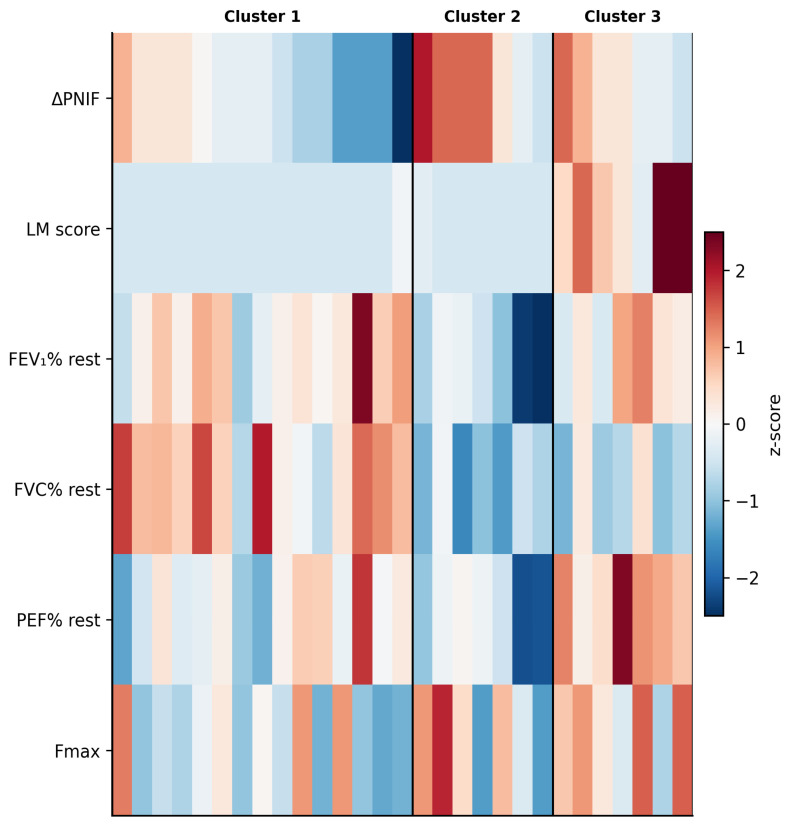
Heatmap of z-scored values for six PCA input variables (*n* = 29). Participants sorted by cluster then descending ΔPNIF. Contrary to our a priori hypothesis, ΔPNIF and LM score do not show a consistent joint gradient across clusters: Cluster 3 combines the highest LM scores with high, not low, ΔPNIF.

Ward’s hierarchical clustering of PC1 and PC2 scores yielded a three-cluster solution (silhouette coefficient = 0.38, indicating only modest cluster separation). Cluster characteristics are summarised in Table 4 (see also Figure S2); contrary to our a priori hypothesis, no cluster combined reduced ΔPNIF with an elevated LM score (Figure 2).

Cluster 1 (*n* = 15; 52%). Moderate exercise-induced decongestion (ΔPNIF: +15.3 ± 15.6 L/min); supranormal spirometry (FEV_1_%: 120.5 ± 10.6%; FVC%: 128.9 ± 12.7%; PEF%: 101.9 ± 20.4%); low measured LM score where available (0.2 ± 0.7, *n* = 9 with CBCT); World Aquatics score 637.9 ± 102.5 pts.

Cluster 2 (*n* = 7; 24%). The largest mean decongestion response of the three clusters (ΔPNIF: +39.3 ± 17.4 L/min), but the lowest spirometric values (FEV_1_%: 99.7 ± 15.0%; FVC%: 103.4 ± 8.3%; PEF%: 80.9 ± 25.1%); low measured LM score where available (0.2 ± 0.5, *n* = 4 with CBCT); World Aquatics score 655.6 ± 115.9 pts.

Cluster 3 (*n* = 7; 24%). The highest measured LM score of the three clusters (9.4 ± 7.7, *n* = 7 with CBCT) but, contrary to our a priori hypothesis, also strong, positive decongestion (ΔPNIF: +29.3 ± 12.4 L/min) and supranormal spirometry (FEV_1_%: 119.9 ± 8.7%; PEF%: 129.1 ± 18.4%). World Aquatics score 640.1 ± 125.6 pts; not different from the other two clusters (*p* = 0.94, Table 4).

### 3.5. Correlates of Competitive Performance

Tethered force (Fmax) was the only statistically significant correlate of World Aquatics performance score (Pearson r = 0.560; 95% CI: 0.247–0.760; *p* = 0.002; r^2^ = 0.31; *n* = 29), explaining ~31% of performance variance. No spirometric variable (resting or post-sprint) correlated significantly with World Aquatics score (all |r| < 0.12; *p* > 0.06). ΔPNIF showed a positive but non-significant trend (r = 0.349; *p* = 0.068). Neither PC1 nor PC2 from the six-variable PCA (Section 3.3) correlated significantly with World Aquatics score (PC1: r = −0.27, *p* = 0.16; PC2: r = −0.08, *p* = 0.68).

## 4. Discussion

**Figure 3:**
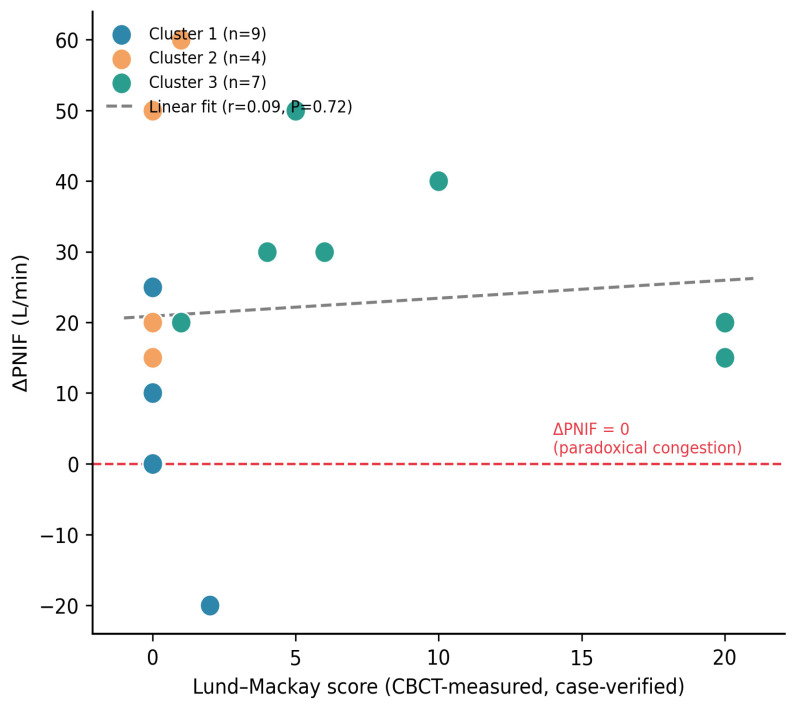
LM score vs. ΔPNIF—CBCT subgroup (*n* = 20). Reference line: ΔPNIF = 0 (red dashed; paradoxical congestion threshold). Spearman ρ = +0.32, *p* = 0.17 (not statistically significant).

This study applied PCA and hierarchical clustering to combined sinonasal, spirometric, and exercise-physiological data in competitive swimmers. Contrary to our a priori hypotheses, three main observations emerge. First, CBCT-derived LM score and post-exercise ΔPNIF were not significantly correlated in this cohort (Spearman ρ = 0.32, *p* = 0.17; Figure 3), in the opposite direction from what we had hypothesised. Second, while PCA and clustering identified structure in the combined dataset, the resulting components and subgroups were not organised along a clean “mucosal-reactivity vs. ventilatory-reserve” axis: LM score loaded with Fmax rather than with ΔPNIF, and the subgroup with the highest LM score also showed the highest, not the lowest, post-exercise decongestion. Third, we found no basis for a discrete “dysfunctional” subgroup, nor for a predictive model combining ΔPNIF and LM score, in this cohort.

Because post-exercise nasal decongestion and bronchial dilation are mediated by different receptor systems (α-adrenergic vasoconstriction in the nasal mucosa versus β_2_-adrenergic bronchial smooth muscle relaxation) [12,28], it remains biologically plausible that sinonasal and ventilatory responses to exercise could dissociate in some athletes. Our data, however, do not provide evidence that CBCT-derived LM score identifies swimmers in whom this dissociation occurs; if such a subgroup exists, it was not detected in the present sample. The non-adrenergic, non-cholinergic (NANC) system, a third branch of autonomic innervation alongside the sympathetic and parasympathetic systems, provides a further candidate mechanism worth considering for the small number of swimmers who showed paradoxical or absent post-exercise nasal decongestion (Section 3.2): NANC neuropeptides such as vasoactive intestinal peptide and substance P modulate nasal mucosal blood flow and glandular secretion independently of classical adrenergic tone, and dysregulation of this pathway has been implicated in non-allergic rhinopathy [29]. Whether NANC-mediated mechanisms contribute to the paradoxical decongestion observed here could not be tested with the present measures and would require targeted pharmacological or neurophysiological assessment.

The magnitude of exercise-induced bronchodilation observed in this cohort—mean ΔFEV_1_ of +9.6 pp and effect size of 0.84—is considerably larger than values typically reported in healthy populations, where EIBd prevalence is approximately 54% [30] and FEV_1_ increases rarely exceed 6%. The high prevalence in swimmers (83%) likely reflects the supramaximal catecholamine surge elicited by a 30 s all-out anaerobic tethered sprint [30]. This adaptive, β_2_-mediated dilatory response is mechanistically and clinically distinct from exercise-induced bronchoconstriction (EIB), the airway-narrowing phenomenon more commonly screened for in athletes [31]; EIBd itself has received comparatively little attention in swimmers specifically, despite growing recognition of exercise-related airway changes across elite athletic populations generally [32,33]. Adolescent elite swimmers show a similarly high combined burden of EIB and rhinitis [34], suggesting that concurrent upper- and lower-airway assessment may be warranted earlier in an athlete’s competitive career than is currently standard practice.

Neither PC1 nor PC2 from this PCA correlated significantly with World Aquatics performance score (PC1: r = −0.27, *p* = 0.16; PC2: r = −0.08, *p* = 0.68), consistent with the absence of a significant correlation between resting spirometry and performance (Section 3.5). ΔPNIF alone showed a positive but non-significant trend with performance (r = 0.349, *p* = 0.068). Tethered force (Fmax) remained the only significant correlate of World Aquatics performance score (r = 0.560, *p* = 0.002), reinforcing the importance of propulsive force generation, rather than ventilatory or sinonasal measures, for competitive performance in this cohort.

Several limitations must be acknowledged. The sample size of 29 participants (20 with CBCT) is modest relative to the number of multivariate methods applied (PCA, hierarchical clustering, and ANOVA), and all reported components and clusters should be read as exploratory rather than confirmatory. CBCT was performed only in participants with a clinical indication, so the measured LM scores are not a random sample of the cohort; median imputation for the remaining participants was intended to be conservative (see Section 2.8). The cross-sectional, single-occasion design means that it is unknown whether repeated testing of the same swimmers would reproduce the cluster structure reported here; longitudinal replication would be required before any post-exercise PNIF/LM-based grouping is treated as a stable, clinically meaningful category. The cross-sectional design further precludes causal inference. Post-exercise spirometry was measured within 3 min of the sprint; because exercise-induced bronchoconstriction can peak later, typically 5–10 min post-exercise, this timing window may have limited detection of delayed bronchoconstrictive responses, and our spirometric findings should be interpreted as characterising the early, not the full, post-exercise interval. Finally, the reproducibility of post-exercise nasal vasoreactivity across repeated sprint sessions [35] requires further investigation. The tethered sprint protocol used to derive Fmax is, however, a well-validated tool for assessing speed- and strength-related determinants of swimming performance [36,37,38], supporting its use here as a stable, ecologically valid performance correlate independent of the sinonasal/ventilatory findings discussed above.

## 5. Conclusions

In this cohort of competitive swimmers, CBCT-derived Lund–Mackay score and post-exercise ΔPNIF were not significantly associated, and hierarchical clustering did not identify a subgroup combining elevated sinus mucosal burden with reduced nasal decongestion. Resting spirometry remained supranormal and did not differ across the ventilatory subgroups identified, consistent with the view that resting spirometry alone does not capture post-exercise nasal or ventilatory dynamics in this population. Post-exercise PNIF and CBCT-derived LM score should each continue to be regarded as informative, independent measures of nasal function in athletes, but our data do not support combining them into a validated screening threshold or diagnostic phenotype without further, prospectively verified evidence. Tethered force (Fmax) remains the strongest correlate of competitive performance identified in this cohort and merits further investigation as a training-relevant outcome, independent of upper-airway findings. For clinical practice, our findings caution against inferring an athlete’s post-exercise ventilatory or nasal function from structural imaging (CBCT/LM score) or from resting spirometry alone: neither reliably tracked the other in this cohort. Until a validated composite marker is available, we suggest that sports-medicine clinicians continue to assess post-exercise PNIF and sinonasal imaging findings as separate, complementary sources of information—each potentially informative in its own right—rather than assuming that one predicts the other or that either can substitute for a full clinical evaluation of an athlete with respiratory symptoms.

## Figures and Tables

**Table 1:** Participant characteristics (*n* = 29; mean ± SD).

Variable	All (*n* = 29)	Male (*n* = 18)	Female (*n* = 11)	*p*
Age (yr)	20.7 ± 2.8	20.7 ± 3.2	20.5 ± 2.1	0.83
Height (cm)	181 ± 7	185 ± 5	174 ± 4	<0.001
PNIF rest—T3 (L/min)	141.4 ± 50.5	154.7 ± 54.1	119.5 ± 36.5	0.064
PNIF post-sprint—T4 (L/min)	165.9 ± 56.1	183.6 ± 55.2	136.8 ± 46.4	0.020
ΔPNIF (L/min)	+24.5 ± 18.0	+28.9 ± 16.3	+17.3 ± 19.2	0.093
FVC% predicted (rest)	118.1 ± 15.8	114.5 ± 16.1	123.7 ± 15.1	0.12
FEV_1_% predicted (rest)	115.3 ± 14.2	114.1 ± 11.5	117.5 ± 18.2	0.32
PEF% predicted (rest)	103.4 ± 26.6	104.5 ± 28.5	100.2 ± 25.4	0.66
Fmax (N)	207.6 ± 49.5	236.9 ± 37.9	159.5 ± 18.2	<0.001
World Aquatics score (pts; *n* = 28)	642.7 ± 107.6	654.7 ± 104.9	623.2 ± 114.0	0.45
DNS present, *n* (%)	25 (86%)	16 (89%)	9 (82%)	0.61
LM score (CBCT subgroup; *n* = 20)	3.5 ± 6.3	4.8 ± 7.1	0.3 ± 0.8	0.15

DNS, deviated nasal septum; LM, Lund–Mackay score; PNIF, peak nasal inspiratory flow; Fmax, peak tethered force. *p* = independent samples *t*-test (male vs. female).

**Table 2:** Ventilatory response to 30 s tethered sprint (*n* = 29; mean ± SD).

Variable	Rest	Post-Sprint	Δ (pp)	*p*	Cohen’s d
PNIF (L/min)	141.4 ± 50.5	165.9 ± 56.1	+24.5	<0.0001	1.36
FVC% predicted	118.1 ± 15.8	120.4 ± 16.2	+2.4	0.034	0.40
FEV_1_% predicted	115.3 ± 14.2	125.0 ± 15.8	+9.6	<0.0001	0.84
FEV_1_/FVC% predicted	100.1 ± 13.3	105.5 ± 8.9	+5.4	0.014	0.50
PEF% predicted	103.4 ± 26.6	118.8 ± 28.1	+15.4	0.004	0.59
FEF_25–75_% predicted	123.0 ± 16.2	125.6 ± 19.2	+2.4	0.130	0.30

pp, percentage points; d, Cohen’s d effect size.

**Table 3:** PCA component loadings and explained variance.

Variable	PC1 (34.8%)	PC2 (28.2%)
ΔPNIF	−0.41	−0.36
LM score	−0.10	+0.53 *
FEV_1_% (rest)	+0.65 *	−0.12
FVC% (rest)	+0.36	+0.44
PEF% (rest)	+0.51 *	−0.43
Fmax	−0.10	−0.44
Eigenvalue	2.16	1.76
Cumulative variance (%)	34.8%	63.1%

* Loadings ≥ |0.50|. LM score imputed at cohort median (0) for non-CBCT participants (*n* = 9); allergic rhinitis status was not included as a PCA input in this analysis (Section 2.8). LM, Lund–Mackay score; PNIF, peak nasal inspiratory flow; Fmax, peak tethered force.

**Table 4:** Cluster characteristics (mean ± SD unless otherwise stated).

Variable	Cluster 1 (*n* = 15)	Cluster 2 (*n* = 7)	Cluster 3 (*n* = 7)	*p*
ΔPNIF (L/min)	+15.3 ± 15.6	+39.3 ± 17.4	+29.3 ± 12.4	0.006
FEV_1_% rest	120.5 ± 10.6	99.7 ± 15.0	119.9 ± 8.7	0.001
FVC% rest	128.9 ± 12.7	103.4 ± 8.3	109.4 ± 9.6	<0.001
PEF% rest	101.9 ± 20.4	80.9 ± 25.1	129.1 ± 18.4	0.001
LM score (measured subset) ‡	0.2 ± 0.7	0.2 ± 0.5	9.4 ± 7.7	0.002
World Aquatics (pts)	637.9 ± 102.5	655.6 ± 115.9	640.1 ± 125.6	0.940
PC1 score	+0.67 ± 1.12	−1.91 ± 0.71	+0.48 ± 0.80	<0.001
PC2 score	+0.84 ± 0.53	−0.05 ± 1.19	−1.75 ± 0.63	<0.001

ANOVA with Tukey’s post hoc test or the Kruskal–Wallis test was used when the normality assumption was not met. ‡ CBCT subgroup: *n* = 20; Cluster 1: *n* = 9; Cluster 2: *n* = 4; Cluster 3: *n* = 7. LM, Lund–Mackay score. ‡ Indicates that the LM score was measured only in the CBCT subgroup.

## Data Availability

The data presented in this study are available on request from the corresponding author due to institutional data protection requirements and participant confidentiality.

## Supplementary Materials

The following supporting information can be downloaded at: https://www.mdpi.com/article/10.3390/jcm15155868/s1. Figure S1: Whole-group ventilatory response to 30-s tethered sprint (paired *t*-test, *n* = 29). Figure S2: Phenotype-specific distributions of key variables (*n* = 29); Figure S3: Paired rest vs. post-sprint changes (each line = one swimmer).
